# Predictors of self-medication with herbal remedies during pregnancy based on the theory of planned behavior in Kashan, Iran

**DOI:** 10.1186/s12906-021-03353-8

**Published:** 2021-08-17

**Authors:** Zahra Karimian, Zohreh Sadat, Bahareh Afshar, Maryam Hasani, Marzieh Araban, Mahbubeh Kafaei-Atrian

**Affiliations:** 1 Department of midwifery, Faculty of Nursing and Midwifery, Kashan University of Medical Sciences, Kashan, Iran; 2 Trauma Nursing Research Center, Kashan University of Medical Sciences, Kashan, Iran; 3 Ms.c of midwifery counseling, Student Research Committee, School of Nursing and Midwifery, Iran University of Medical Sciences, Tehran, Iran; 4 Department of midwifery, School of Nursing and Midwifery, Lorestan University of Medical Sciences, Khorramabad, Iran; 5 Department of Health Education and Promotion, Public Health School, Ahvaz Jundishapur University of Medical Sciences, Ahvaz, Iran; 6 Department of midwifery, Faculty of Nursing and Midwifery, Kashan University of Medical Sciences, Kashan, Iran

**Keywords:** Herbal medicine, Pregnancy, Theory of planned behavior

## Abstract

**Background:**

The incidence of application of medicinal herbs during pregnancy has increased significantly among women over the past years; however, *the* safety and efficacy of medicinal herbs during pregnancy are still unclear. The aim of the present study was to categorize the predictors of self-medication with herbal remedies during pregnancy based on the theory of planned behavior (TPB).

**Methods:**

The present descriptive-analytical study was conducted on 300 pregnant women referred to Kashan health center to receive prenatal care services in 2020. The study participants were randomly selected using stratified random sampling with proportional allocation. The data collection tool was a two-part researcher made questionnaire. The first part of the questionnaire included demographic information, midwifery information, and questions related to women’s awareness about herbal medicine. The second part of the questionnaire was designed based on the theory of planned behavior including attitudes, perceived behavioral control, subjective norms, intention, and behavior performance. Data were analyzed using descriptive statistics, regression analysis, and SPSS version 18.0.

**Results:**

The mean age of participants was 28. 7±5.4 years (range, 15–45 years), the majority were housewives (88.3%) and had secondary education (39.3%). A total of 164 women (57. 1%) used medicinal herbs during pregnancy. The individual’s attitude towards herbal medicines consumption, subjective norm, and perceived behavioral control was correlated with behavioral intention (*P* < 0.05). Similarly, subjective norms were the most predictor of using herbal medicine among pregnant women (*P* < 0.05).

**Conclusion:**

The findings revealed that more than 50 % of pregnant women used medicinal herbs during pregnancy. The present study showed that the individual’s attitude towards herbal medicines consumption, subjective norm, and perceived behavioral control was correlated with intention of herbal medicine use among pregnant women. Likewise, subjective norms were the most predictor of herbal medicine use among pregnant women**.** The TPB should be addressed in planning health education programs and modifying health behaviors, including self-medication, especially during pregnancy.

## Background

The terms “complementary medicine” or “alternative medicine” refer to a broad set of health care practices that are not part of that country’s own tradition or conventional medicine and are not fully integrated into the dominant health-care system [1].

The use of herbal medicine has increased across the globe [2–4], about 65–80% of the world’s population use some form of herbal medicinal products [3]. The prevalence and pattern of herbal medicines use in different regions of Iran is about 19. 2–90. 2%, different studies report different rates [5–9]. Ginger, mint syrup, and chamomile are among the medicinal herbs which have been reported to be consumed during pregnancy [10, 11].

Herbal medicines have valuable medicinal properties; however, adverse reactions and side-effects of herbal medicine have been reported [12, 13]. Therefore, herbal medicine consumers should be aware of interactions and side effects of herbal medicines and herbal dietary supplements [14]. Many patients believe that herbal medicines are safe [15, 16]; however, herbal remedies like chemical drugs can cause many side effects [17, 18].

Many studies have shown that women are more likely to use medicinal plants and traditional medicine [19, 20]. Some conditions during pregnancy such as nausea, vomiting, and constipation can lead to the consumption of herbal medicine [21–23]. Taking herbal medicine during pregnancy can lead to serious consequences for mother and the fetus [24, 25]. Herbal medicine consumption should be monitored during pregnancy and pregnant women need to ensure they are getting appropriate and safe herbal remedies [26–28].

Studying the herbal medicine use and predictors during pregnancy supports the health of mother and her unborn baby [29]. Several professionals have taken positive stands to the dynamic nature of health education and health behavior models in the context of health-promoting behaviors and their predictors [30]. Therefore, choosing an appropriate model for health education is the first step of any preventive health behavior program [31]. One of the theories that has been successfully used to identify and modify health behaviors, including self-medication, is the theory of planned behavior [32].

The theory of planned behavior (TPB) has been extensively utilized to explain health-related behavior. TPB suggests that the proximal determinants of individual behavior are behavioral intention and perceived behavioral control. Intention is determined by subjective norms, attitude, and perceived behavioral control. Attitude toward certain behavior is defined as the positive or negative evaluation of the person towards a behavior. Subjective norms refer to the perceived social pressures and expectations concerning whether an individual engages in a given behavior or not. Perceived behavioral control refers to individual perception (internal/external) of the ease or difficulty of performing a certain behavior [31]. On the other hand, TBP proposes that attitude, subjective norm, and perceived behavioral control trigger behavioral intention. Both intention and perceived behavioral control contribute directly to the prediction of behavior [31].

Although TPB has been widely used for the prediction of health-related behaviors, traditional Chinese medicine utilization, supplement-taking behavior, and intention to purchase complementary and alternative medicine, there is insufficient evidence on self-medication with herbal medicines in pregnant women [33–35]. TPB has the potential to identify the predictors of health-related behavior among pregnant women [36].

Taking herbal medicine during pregnancy can lead to serious consequences for both mother and the fetus. Therefore, self-medication with herbal medicine during pregnancy should be avoided. A high prevalence rates of self-medication with herbal medicine during pregnancy with behavioral beliefs related to self-medication with medicinal herbs have been reported in the literature. However, the prevalence rates and predictors of self-medication with medicinal herbs during pregnancy have not been studied in Kashan (a county in the center of Iran with a rich source of medicinal plants). The present study was conducted to examine the predictors of self-medication with herbal remedies during pregnancy based on the theory of planned behavior.

## Methods

### Study design and setting

The present study was a descriptive/analytical cross-sectional study. A total of 300 pregnant women referred to Kashan health center to receive prenatal care services in 2020 were selected for the study. Inclusion criteria included being pregnant (at any age of pregnancy), willingness to participate in the study, being Iranian, and not having any chronic diseases. Participants who did not meet the eligibility criteria were excluded from the study.

### Sample and data collection

The study participants were randomly selected from all health care centers of Kashan county, using stratified random sampling with proportional allocation. First, the entire population was divided into five categories (strata) based on the geographical location and livelihood level. Then, a random sample from each stratum (subgroup) was taken on the basis of health care coverage, from which the subgroup was randomly selected using a random number table. To address this, the medical record information of pregnant women referred to health care centers from April to July 2020 was screened using random number table. Women were screened for their eligibility and invited to participate in the study via phone call for completing the questionnaire. The questionnaire was completed by three trained interviewers. Interview was used for data collection. The interview took place in one face-to-face interview session. To encourage the participants, they were given an online exercise program during pregnancy. The sample size was considered according to other studies [8] and the sample size formula: $n=\frac{\left({z}_{1-\frac{\propto }{2}}\right)2\times p\left(1-p\right)}{d^2}$) where α: 5%, d: 6%, p: 50%. Finally, a total of 294 pregnant women (with considering 10% for drop sample) were selected for the study.

Data collection tools included a researcher-made questionnaire including questions about demographic information (13 items), midwifery information (13 items), and awareness (7 items, such as “herbal medicine can affect the fetus”). Awareness questions were scored on a true or false basis. The total score ranged from 0 to 7, with a higher score indicating better awareness. The reliability of the scale was measured by Cronbach alpha and it was satisfactory (0.81).

The second part of the questionnaire was designed based on the theory of planned behavior including: 1- Attitudes towards the use of medicinal herbs during pregnancy and post-delivery (7 items, e.g., in my opinion, the use of herbal medicines is .... absolutely useful / absolutely harmful, the total score ranged from 7 to 49, with a higher score indicating positive attitudes); The reliability of the scale was measured by Cronbach alpha and it was satisfactory (0.89).

2- Subjective norms (4 items, e.g., most people who are important to mee suggest that I can use herbal medicine and traditional medicine .... Strongly agree/strongly disagree, the total score ranged from 4 to 16, with a higher score indicating being more influenced by people to practice behavior); The reliability of the scale was measured by Cronbach alpha and it was satisfactory (0.82).

3- Perceived behavioral control (2 items, choice of perceived behavioral control to reasoned action, e.g., despite the special conditions of pregnancy (nausea, vomiting), I can refrain from taking herbal and traditional medicines (the total score ranged from 2 to 4, with a higher score indicating more perceived behavioral control). The reliability of the scale was measured by Cronbach alpha and it was satisfactory (0.74).

4- Intention (2 items, e.g., I plan to take herbal medicine if I experience health problems during pregnancy, strongly agree/strongly disagree, the total score ranged from 2 to 4, with higher scores indicating greater intention). The reliability of the scale was measured by Cronbach alpha and it was satisfactory (0.80).

5- Behavior performance (22 items, five-point Likert scale including always, often, sometimes, rarely, never, the total score ranged from 22 to 110, with higher scores indicating more tendency toward herbal medicines during pregnancy). The reliability of the scale was measured by Cronbach alpha and it was satisfactory (0.89).

The validity of the questionnaire was determined using content validity ratio (CVR) and content validity index (CVI). To establish content validity, 10 academicians including experts on health education, public health, midwifery, and obstetrics were asked to categorize the items into three categories of “necessary,” “beneficial but not necessary,” and “not necessary. According to the Lawche’s table, the items with CVR > 0.62 were remained in the questionnaire. For CVI estimation, the experts were asked to score each item based on relevance and clarity using a 4-point Likert type scale. Items were regarded as clear and relevant if they obtained values ≥0.79. Finally, after two rounds, the content validity ratio (CVR) and content validity index (CVI) were calculated, which were both equal to one.

### Ethical considerations

The questionnaires were completed through face-to-face interviews by trained midwifery experts. All participants were informed about the aims of the study. Participants were assured that participation in the study is voluntary and refusal to participate will comprise no penalty or loss of maternity care services. Moreover, in order to appreciate the participants, a contact number was provided to the research units to answer possible questions during pregnancy. This study is the result of a research project No: 98000262 and approved by Ethics Committee of Kashan University of Medical Sciences (Registration No: IR.KAUMS.NUHEPM.REC.1399.023).

### Statistical analysis

Descriptive statistics were used to describe the basic features of the data. T-test was used to compare the means of two groups. Chi-square test was used to compare categorical variables. ANOVA test was used to analyze association between socio-demographic characteristics and construct of the theory of planned behavior. Multiple linear regression analysis was used to determine the factors affecting the use of herbal medicines. All models and variables were checked for multicollinearity with the variance inflation factor (VIF) statistics and no adverse consequences was reported. To verify the TPB model, the relationship between demographic variable, the constructs of the theory of planned behavior, and dependent variables (self-medication behavior) was examined. *p*-value of less than 0.05 was considered significant in the analyses. The final model was identified using backward elimination method. Data were analyzed using SPSS (SPSS Inc., Chicago, Ill., USA) version 18.0.

## Results

The response rate of participants was 100%. The mean age of participants was 28. 7±5.4 years (range, 15–45 years), the majority were housewives (88.3%) and had secondary education (39.3%). The most of participants (65. 7%) reported their economic status as fair. The incidence and the percentage of pregnancies were between 1 and 6 and the mean number of pregnancies were 2. 2± 1.3. 164 of women (57. 1%) used medicinal herbs during pregnancy. The association between socio-demographic characteristics and construct of the theory of planned behavior is presented in Table 1. The use of herbal remedies was higher among younger women.

**Table 1:** The relationship between the TPB constructs and demographic variables

Variables	Group	Awareness	Attitude	Subjective norms	Behavioral intention	Behavioral control	Behavior Performance (herbal self-medication)
Age Mean ± SD 28. 7±5/4	20<	15±0. 6	24.5± 1. 6	16. 2±0. 6	4. 1±0.3	9.4 ± 0.3	**105±** 1**.** 7
	21-30	15. 1±0.3	23±0.4	16. 6±0.3	4. 2±0.09	8. 7±0. 1	**101±** 1**.** 2
	31-40	14.3 ± 0.3	23.06 ± 0.4	16.04 ± .03	4.3 ± 0. 1	9±0. 1	**101±** 1**.3**
	40>	20± 1	27.5 ± 0.5	16±0.5	4.5 ± 0.5	8±0. 1	**100±** 1**.** 1
	*p*-value*	0.5	0. 2	0.01	0. 9	0.4	**0.** 9
Occupation status	Employed	13.5 ± 0. 9	24. 1± 1. 1	15.5 ± 0. 8	4 ± 0.3	9.3 ± 0. 6	**100 ± 4.07**
	Housewife	14.4 ± 0.3	22. 9±0.3	16. 6±0. 2	4.3 ± 0. 1	8. 9±0. 1	**101 ± 0.** 9
	*p*-value**	0.4	0. 8	0. 1	0. 2	0.3	**0.3**
Education	Illiterate and elementary	13. 1± 1.03	24. 2± 1. 6	17.4 ± 0. 9	4.3 ± 0. 2	9±0.5	**101±** 2**.** 9
	Middle School	14. 1±0. 6	23.5 ± 0.5	16.4 ± 0.4	4. 1±0. 1	8. 6±0.3	**104±** 1**.** 2
	High school	14. 7±0.4	23.5 ± 0. 6	16. 7±0.3	4.3 ± 0. 1	8. 7±0. 2	**100±** 1**.5**
	University Education	14. 2±0.5	22. 1±0. 7	16.05 ± 0.4	4.3 ± 0. 1	9.4 ± 0. 2	**101±** 2
	*p*-value*	**0.** 1	**0.** 6	**0.** 1	**0.03**	**0.3**	**0.5**
Economic status	Good	13.4 ± 0. 6	21. 6±0. 8	16. 7±0. 6	4.4 ± 0.3	10.03 ± 0.4	**98.** 6**±** 2**.** 2
	Fair	14.3 ± 0.3	23.5 ± 0.4	16.4 ± 0. 2	4. 2±0. 1	8. 7±0. 1	**101±** 1**.** 2
	Poor	14.4 ± 0. 6	23.3± 1. 1	16. 1±0. 7	4.5 ± 0. 2	8. 8±0.4	**103±** 1**.** 7
	*p*-value*	0.3	0. 1	0. 2	0. 1	0. 1	**0.** 6

*Anova, **t-test

Analysis of variance (ANOVA) indicated a significant difference between subjective norms and age (*P* = 0.01). There was a significant difference between the behavioral intention score and education (*P* = 0.03). No significant difference was found between the other socio-demographic variables and construct of TPB (*P* > 0.05).

According to the results of Table 2, each of the TPB variables was correlated with together, expect Subjective norms with Perceived behavioral control, (*P* < 0.05).

**Table 2:** Results of the correlation analysis between each of the TPB variables

variable	intentions to use	Perceived behavioral control	Subjective norms	Attitude	behavior performance
**intentions to use**	1				
**Perceived behavioral control**	0.27*	1			
**Subjective norms**	−0.52*	**−0.08**	1		
**Attitude**	0.47*	**0.** 24*	−.04*	1	
**behavior performance**	0.30*	0.18*	−0.47*	0.32*	1

*Correlation is significant (*P* **< 0.05)**

According to the results of Table 3, the individual’s attitude towards herbal medicines consumption, subjective norm, and perceived behavioral control was correlated with behavioral intention (*P* < 0.05). R^2^ = 0. 38

**Table 3:** The correlation between behavioral intention and herbal medicine use during pregnancy

Variables and constructs	Std. Error	Β	T	Sig.
**Attitude**	0.01	0.30	4.83	0.00
**Subjective norms**	0.02	−0.32	-5. 2	0.00
**Perceived behavioral control**	0.05	0.13	2.3	0.02

According to the results of Table 4, herbal self-medication during pregnancy was correlated with subjective norms (*P* < 0.05), R^2^ = 0.27.

**Table 4:** Correlation between self-medication with medicinal herbs and subjective norms among pregnant women

Variables		Std. Error	Β	t	Sig.
**Herbal self-medication** **during pregnancy**	Behavioral control	.47	−.02	-. 35	.72
	Awareness	. 15	.13	2.09	.03
	Attitude	.13	.08	1.19	. 23
	Subjective norms	.21	−.37	− 4.98	.00
	Behavioral intention	.55	.13	1.71	.08

There was no significant linear relationship between the constructs of the theory of planned behavior and each of the demographic variables (*P* > 0.05). Behavioral constructs of TPB were not associated with herbal self-medication in the postpartum period (P > 0.05).

## Discussion

The current study showed that more than half of pregnant women used herbal medicines. The use of herbal medicine during pregnancy has been reported differently in different parts of the world. A multinational study was carried out in Europe, North and South America, and Australia (2011–2012). A total of 9,459 women from 23 countries were participated in the study, of which 28. 9% used herbal medicines during pregnancy (ranged from 4.3% in Sweden to 69% in Russia) [37]. In another study, the prevalence of herbal medicines use among pregnant women in Gulu district (2016) was 20% [38]. The reasons for this variations could be explained due to different regions,, cultural differences,, customs, and research methods. But the reason of the high prevalence of herbal consumption in Iran can mainly be attributed to easy access and insufficient education in herbal medicines.

The present study showed that the individual’s attitude towards herbal medicines consumption, subjective norm, and perceived behavioral control was correlated with behavioral intention (taking herbal medicines). In most behavioral domains, a strong intention-behavior relationship is recognized [39, 40]. Dzulkipli et al. found that subjective norm and perceived behavioral control was correlated with behavioral intention (R^2^ = 0.39) which was consistent with the results of the present study (R^2^ = 0. 38) [41]. Conner et al. suggested that subjective norm and attitude was correlated with behavioral intention (R^2^ = 0.70) [35].

Negative coefficient suggests that as the subjective norms variable increases, the behavior performance variable tends to decrease. The coefficient value signifies how much the mean of the behavior performance variable changes given a one-unit shift in the subjective norms variable while holding other variables in the model constant. This shows that the tendency to perform behavior is not only influenced by individual, but also is controlled by abstract norms. Therefore, in behavioral modification the individual subjective norms need to be taken into consideration.

The present study showed that younger women were more likely to use herbal medicines. The results of this study also showed that subjective norms had a significant effect on the application of herbal medicine among pregnant women, i.e., if a woman feels that taking herbal medicine during pregnancy is acknowledged by family members and friends, she is more likely to use it. This indicates that subjective norms should be deemed as exogenous variable and need to be addressed in the prenatal education programs for pregnant women. Studies suggested that family members and close relatives can significantly influence the use of herbal medicine among pregnant women [42, 34]. However, in the Afshary et al.’s study, behavioral control was the prominent factor that influenced the practice of herbal medicine [9].

Afshary et al., 2015 stated that behavioral control following attitude were the leading predictors of herbal medicine use which was inconsistent with the results of the present study. The discrepancy in the findings of two studies is likely due to dissimilar study samples used in each study [9]. Studies have shown the positive influence of family and friends in self-medication by herbal medicine during pregnancy than other stages [42, 34]. Considering the results of this study and the importance of subjective norms, the role of relatives in self-medication should be considered in prenatal education program.

The role of health care provider in the management of self-medication has been confirmed [43]. Therefore, providing comprehensive training programs for healthcare providers (nurses/midwives) about medicinal plants and natural remedies particularly during pregnancy is recommended.

### Strengths and limitations of the study

The main strength of the present study is a large and properly selected sample size. This study, however, is subject to several limitations. First, patients refused to recount the use of herbal medicines due to the cultural context of Kashan and the disrespectful behavior of some health care setting. To solve this limitation of the study, it was tried to explain the objectives of the research to the participants correctly and to emphasize that the results of the study will have no effect on their treatment process. Another limitation of the study was reliance on the self-report for measurement of the studied variables which can cause a particular type of measurement bias. It should be noted that the finding of this study cannot be generalized to *other study* settings including rural setting.

Considering the cross-sectional design of the study, the causal relationship could not be inferred. Further researches concerning the impact of educational programs based on the theory of planned behavior are recommended.

Finally, according to the results of this study, the theory of planned behavior should be considered in designing interventions. Since subjective norms are correlated with herbal medicines use during pregnancy, education of relatives and friends should be considered in pregnancy education program. In addition, in any pregnancy education program the pregnant women should be well oriented on the impact of self-medication with herbal medicine on mother and child’s health during pregnancy and postpartum.

## Conclusion

The present study showed that the individual’s attitude towards herbal medicines consumption, subjective norm, and perceived behavioral control was correlated with intention to use herbal medicine among pregnant women. Also, subjective norms were the most predictor of using herbal medicine among pregnant women**.** Constructs of TPB should be considered in planning health education programs by antenatal educators to address self-medication with medicinal herbs during pregnancy.

## Data Availability

The data used and analyzed in this study are available from the Corresponding author on reasonable request.
